# Rare GATA6 variants associated with risk of congenital heart disease phenotypes in 200,000 UK Biobank exomes

**DOI:** 10.1038/s10038-021-00976-0

**Published:** 2021-09-07

**Authors:** Simon G. Williams, Dominic J. F. Byrne, Bernard D. Keavney

**Affiliations:** 1 Division of Cardiovascular Sciences, Faculty of Biology, Medicine and Health, The University of Manchester, Manchester, UK; 2 Manchester University NHS Foundation Trust, Manchester Academic Health Science Centre, Manchester, UK

**Keywords:** Genome-wide association studies, Congenital heart defects, Genetics research, Genetic predisposition to disease

## Abstract

Congenital heart disease (CHD) has a complex and largely uncharacterised genetic etiology. Using 200,000 UK Biobank (UKB) exomes, we assess the burden of ultra-rare, potentially pathogenic variants in the largest case/control cohort of predominantly mild CHD to date. We find an association with GATA6, a member of the GATA family of transcription factors that play an important role during heart development and has been linked with several CHD phenotypes previously. Several identified GATA6 variants are previously unreported and their roles in conferring risk to CHD warrants further study. We demonstrate that despite limitations regarding detailed familial phenotype information in large-scale biobank projects, through careful consideration of case and control cohorts it is possible to derive important associations.

Anomalies arising in early embryonic development can result in a range of congenital heart disease (CHD) phenotypes, from complex conditions with multiple defects, such as tetralogy of Fallot (TOF), to comparatively mild phenotypes that may go undiagnosed until later life, such as isolated bicuspid aortic valve (BAV) [1]. Several genes have been associated with CHD risk in previous GWAS and sequencing studies, but studies involving larger numbers of case samples remain needed to facilitate further understanding of what remains a complex and largely uncharacterised genetic etiology.

Here we use exome sequencing data from the first 200,000 samples in UK Biobank (UKB) to assess rare and potentially pathogenic variation associated with increased risk of CHD. Using hospital episode statistics (HES) and primary care diagnostic codes in a classification scheme we previously reported [2], we identified 1354 individuals with CHD phenotypes and classified 179310 individuals as non-CHD controls. Being a subset of the entire UKB cohort, the full assortment of CHD phenotypes included here represents those previously reported [2], albeit with an approximately proportional decrease in numbers, with many individuals containing multiple classifying codes. The most common CHD-related codes were aortic stenosis (*n* = 395), aortic insufficiency (*n* = 278) and evidence of aortic valve replacement (*n* = 270). We inferred that clinically manifest aortic valve disease (AVD) before the age of 65 was highly likely to be due to BAV and classified this subset of cases accordingly. UKB participants with AVD manifesting after age 65 were assigned unknown status. As previously observed, more severe CHD conditions within the case cohort such as atrial septal defect (*n* = 76), ventricular septal defect (*n* = 69) and TOF (*n* = 13) occur infrequently in UKB participants, demonstrating the known ‘healthy cohort bias’ in UKB where more severe disease phenotypes, including CHD, are under-represented [3]. The UKB cohort therefore represents the largest case/control analysis of predominantly mild CHD phenotypes with exome sequencing to date.

We hypothesised that genes contributing to CHD risk have an increased burden of ultra-rare, potentially pathogenic variants in cases compared to controls. We conducted a burden test to identify such variants at the gene level. Variants were first filtered (genotype quality (GQ) ≥ 20; read depth (DP) ≥ 10 for indels and DP ≥ 7 for SNPs) before being annotated with Variant Effect Predictor (VEP) v98. To focus on potentially pathogenic variants, we retained only variants present in <1% of the total UKB samples, with HIGH/MODERATE impact, absent in gnomAD and CADD score ≥ 20. Additionally, to assess potential differences in more common variation between case and control groups, synonymous variants with gnomAD AF > 0.01 were extracted. Qualifying variants (QV) for each analysis were collapsed to the gene level for burden analysis.

Following removal of related individuals and ensuring ancestral similarity between groups using principal component analysis, case and control cohorts were compared. For common synonymous variants, the QQplot of variants indicates no difference between case and control groups (Fig. 1A). Conversely, the QQplot of ultra-rare pathogenic variation shows inflation caused by a small number of genes with significantly increased prevalence in the CHD group (Fig. 1B). A burden analysis of these rare pathogenic QV highlights GATA6 as the gene with the most significant CHD association (OR = 7.66 [95% CI 3.6–14.47]; *p* = 1.60E–06), with 9 unique QV present in 10 CHD samples, and the only significant candidate following Bonferroni correction (*p* = 0.03) (Table 1). As absence from gnomAD represents a stringent threshold, potentially excluding pathogenic variants occurring at a low level, we conducted a subsidiary analysis of variants with gnomAD AF < 0.0001. No gene showed a significant burden of variants at this threshold in CHD cases.

**Fig. 1:**
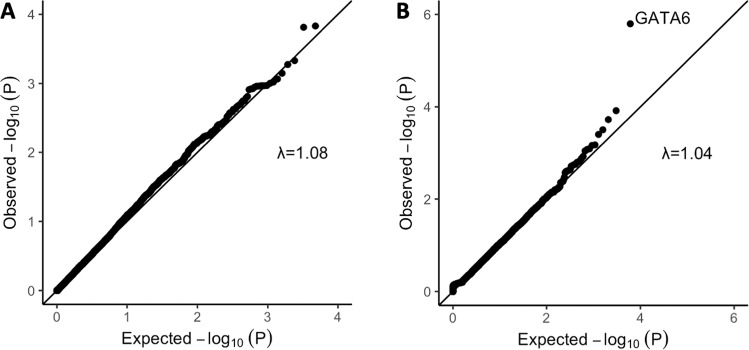
**A** QQ plot of common (gnomAD AF > 0.01) synonymous variants between CHD cohort and controls in autosomes and QQ plot of rare (not present in gnomAD) and **B** potentially pathogenic (HIGH/MODERATE impact and CADD ≥ 20) variants between CHD cohort and controls.

**Table 1:** Leading candidates from the case/control burden analysis.

Gene	Case prevalence (%)	Control prevalence (%)	Odds ratio	*P* value	p.adjust (Bonferroni)
GATA6	0.74	0.10	OR = 7.66 (95% CI 3.6–14.47)	1.60E–06	3.24E–02
N4BP2L2	0.44	0.05	OR = 8.4 (95% CI 3–19.01)	1.21E–04	1
ZNF398	0.30	0.02	OR = 15.62 (95% CI 4.02–43.86)	1.89E–04	1
MAMLD1	0.22	0.01	OR = 26.52 (95% CI 4.92–94.03)	3.15E–04	1
PKN1	0.37	0.04	OR = 8.63 (95% CI 2.72–21.07)	3.97E–04	1

GATA6 is a member of a family of zinc-finger transcription factors that play an important role during heart development by regulating cellular differentiation. GATA6 has previously been associated with multiple CHD phenotypes [4–8], the first association described being with persistent truncus arteriosus, though association with BAV has not previously been shown in population genomic data. GATA6 haploinsufficient mice have been shown to have BAV through a proposed mechanism of dysregulated extracellular matrix regulation, and human BAV tissues removed at surgery have lower levels of GATA6 expression than do tricuspid aortic valves [4]. GATA6 has a similar expression pattern to GATA family member GATA4, a well-established CHD-associated gene; a previous GWAS showed association between protein-altering and regulatory common variants near GATA4 and BAV [9]. A recent paper [10] reviewed the literature on the genotypic and phenotypic spectrum of GATA6 in humans, with focus on more severe conditions. Eighty percent of variant carriers reported structural cardiac phenotypes, predominantly atrial or ventricular defects. Additionally, the association of GATA6 with pancreatic anomalies was confirmed. Previously reported CHD-associated missense variants cluster in the DNA-binding domains where they likely severely disrupt function. Mapping the UKB CHD QVs to the GATA6 protein shows no clustering in these domains (Fig. 2), indicating that some GATA6 function may be retained, potentially resulting in the milder CHD phenotypes observed. Of the GATA6 variants identified, 7 are previously unreported (NP_005248.2:p.(Gly6Arg);p.(Gln120Ter);p.(Ser156Gly);p.(Gly250Asp);p.(His258Gln);p.(Pro270Ser);p.(Ser424Ile)) while NP_005248.2:p.(Gly236Cys) and NP_005248.2:p.(Val259Ile) have been reported in ClinVar as variants of uncertain significance in relation to atrioventricular septal defects. Additionally, case samples with GATA6 QVs have no previous history of pancreatic anomalies, including diabetes.

**Fig. 2:**
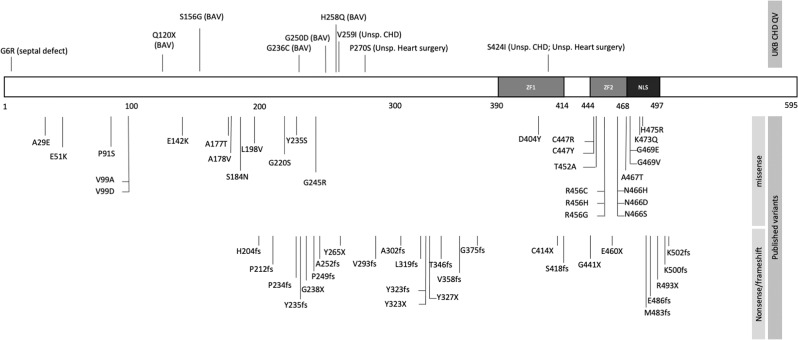
Schematic of GATA6 protein with two DNA-binding zinc-finger domains (ZF1, ZF2) and nuclear localisation sequence (NLS). The location of previously published variants – both missense and nonsense/frameshift – are shown along with the UKB CHD QV. The CHD phenotypes of the individuals with GATA6 qualifying variants are shown.

The UKB resource is suboptimal in terms of additional in-depth phenotypic information or familial history that would be desirable in the clinical study of CHD and more specifically BAV. In the general population, BAV affects around 1% of males and 0.33% of females and is therefore the most common CHD condition [11, 12]. Due to the lack of specific BAV diagnostic codes in these data we broadly grouped AVD-related phenotypes occurring <65 years as ‘inferred BAV’. This identifies an overall prevalence of BAV of 0.4% within this cohort. Misclassification of patients with early degenerative disease of a tricuspid aortic valve as BAV could have occurred as a result of this schema; and if so would have limited the power of our analyses. Despite these limitations our findings indicate that rare variants in GATA6, presumably with a lesser effect on gene function than those causing severe CHD phenotypes, or buffered by other genetic and environmental effects during development, are also associated with minor CHD conditions. Additionally, the absence of other gene candidates in this study confirms the wide heterogeneity of CHD phenotypes where rare variants in single genes only account for a small proportion of cases.

## Data Availability

This research has been conducted using data available from the UKB resource under project 19056.
